# Caspase-14-like Proteases: An Epidermal Caspase and Its Evolutionarily Ancient Relatives

**DOI:** 10.3390/biom15070913

**Published:** 2025-06-22

**Authors:** Leopold Eckhart, Attila Placido Sachslehner, Julia Steinbinder, Heinz Fischer

**Affiliations:** 1 Department of Dermatology, Medical University of Vienna, 1090 Vienna, Austria; attila.sachslehner@meduniwien.ac.at (A.P.S.); julia.steinbinder@meduniwien.ac.at (J.S.); 2 Division of Cell and Developmental Biology, Center for Anatomy and Cell Biology, Medical University of Vienna, 1090 Vienna, Austria; heinz.fischer@meduniwien.ac.at

**Keywords:** caspase, apoptosis, pyroptosis, cornification, pyrin, protein domain, inflammasome, evolution, gene duplication, keratinocyte

## Abstract

Caspases are a family of cysteine-dependent aspartate-directed proteases implicated in programmed cell death. Humans have eleven proteolytically active caspases, namely caspase-1 through -10 and caspase-14. The latter is expressed exclusively in epithelial cells and constitutively resides in its active form in the cornified layer of the human epidermis. Molecular phylogenetics has revealed that caspase-14 belongs to a subfamily of caspases, which also includes caspase-15 and -16. The latter are evolutionarily more ancient than caspase-14 and have been lost in the phylogenetic lineage leading to humans. Here, we review the molecular properties, the species distributions, and the biological roles of caspase-14-like proteases in amniotes. In contrast to the prodomain-less caspase-14, caspase-15 contains a prodomain that is predicted to assume a pyrin fold, and caspase-16 features a prodomain with unique sequence similarity to the catalytic domain. Gene knockout in mice, evolutionary gene loss in aquatic mammals and the association of human *CASP14* mutations with ichthyosis indicate that caspase-14 is associated with the barrier function of mammalian skin. Caspase-15 is able to induce apoptosis in cell culture, but its role in vivo and the role of caspase-16 are currently unknown. We propose directions for research to further characterize caspase-14-like proteases.

## 1. Introduction

Caspases are central regulators and effectors of programmed cell death, pro-inflammatory signaling, cell differentiation and other processes [1,2,3,4,5]. The diversity of functions of caspases is achieved by differences in structures and expression patterns of individual caspases. Molecular phylogenetics has confirmed the common ancestry of all caspases and defined subfamilies of caspases [6,7]. In this review, we summarize the knowledge about a subfamily of caspases, comprising caspase-14, -15 and -16, which are closely related in terms of phylogenetics while representing three distinct types of molecular organization, each of which is unique among mammalian caspases.

Caspases are cysteinyl aspartate proteinases, which means that they depend on a cysteine residue in the catalytic site and cleave proteins after aspartic acid residues [8,9,10]. Importantly, not every aspartic acid residue (D in the one-letter code of amino acids) is a target for caspases because the suitability of cleavage sites depends also on the neighboring residues [11,12,13,14,15]. The preference for sequence motifs varies among caspases, so different proteins and, occasionally, different sites within the same protein are cut by different caspases. Furthermore, the expression of caspase genes leads to catalytically inactive proforms of caspases that depend on dimerization or oligomerization and targeted, often autocatalytic, proteolysis in the linker region between their large and small subunit. Together, the availability of cleavage sites and the activation of specific caspases determine the biological consequences of caspase activities, which range from the proteolytic activation of interleukin-1 family cytokines in the initial phase of inflammation to the destruction of a large part of the cellular proteome in apoptosis [16].

The catalytic activity of caspases is mediated by the so-called caspase domain, which is well defined in the SMART and PFAM protein domain databases (smart00115: CASc, https://smart.embl.de, last accessed on 28 April 2025, pfam00656: peptidase_C14, https://www.ebi.ac.uk/interpro/entry/pfam/PF00656/, last accessed on 28 April 2025) [17,18]. This domain is strictly conserved in caspases but also in catalytically inactive caspase paralogs, such as c-FLIP (cellular Fas-associated protein with death domain (FADD)-like interleukin-1β-converting enzyme-inhibitory protein) [19]. The proteolytic activity requires the presence of the catalytic dyad consisting of a cysteine and a histidine residue [8].

**Figure 1:**
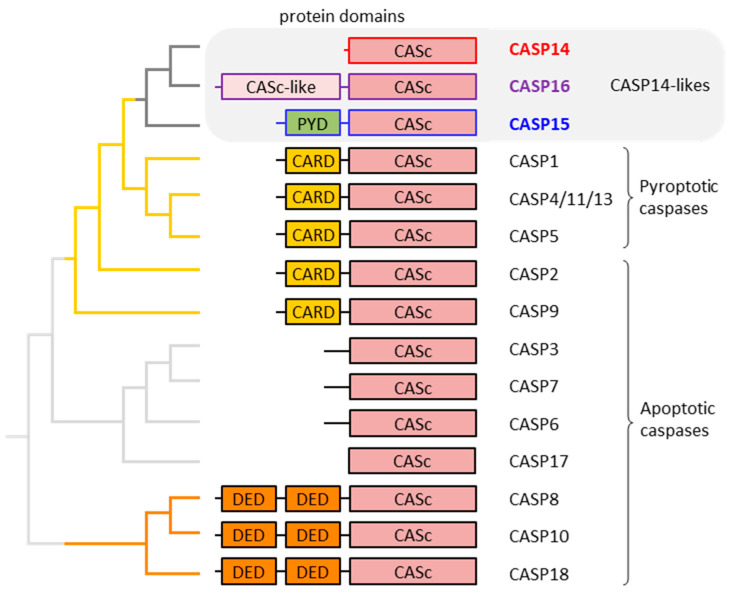
Domain organization and phylogenetic relationship of caspases (CASPs). The organization of protein domains in mammalian caspases is schematically depicted. On the left, the relationships of the caspases are depicted by a phylogenetic tree which is the consensus of published molecular phylogenetics studies [7,32] and the comparative analysis of the exon–intron structure of caspase genes [6]. Catalytically inactive caspase family proteins such as c-FLIP, encoded by *CFLAR* and caspase-12 [33], are not included in this diagram. The phylogenetic position of CASP17 is uncertain. CARD: caspase activation and recruitment domain; CASc: caspase domain; DED: death effector domain; PYD: pyrin domain.

In many but not all caspases, the catalytic domain is preceded by a prodomain. The prodomain of human caspase-1, -2, -4, -5 and -9 consists of a caspase recruitment domain (CARD), whereas two death effector domains (DEDs) form the prodomain in caspase-8 and -10 [5,8]. A CARD is also present in caspase-12 of mammals [20], and DEDs are conserved in caspase-18 [6,21]. Both CARD and DED belong to the superfamily of death domains, which mediate homotypic protein interactions [22,23]. Another type of death domain is the pyrin fold, which, like CARDs and DEDs, is present in regulatory proteins. In non-human caspases, such as caspase-15 [24] and the fish caspases caspy (caspa) and caspy2 (caspb) [25], the pyrin domain is also found in the prodomain. A fly caspase, known as STRICA, contains a serine-threonine-rich prodomain without a defined protein–protein interaction motif [26]. As will be discussed in more detail below, mammalian caspase-16 has a prodomain with sequence similarity to the catalytic (CASc) domain [7]. Other caspases, such as caspase-3, -6 and -7, have relatively short prodomains that are cleaved off during activation [27,28]. Caspase-14 [29,30,31] and caspase-17, which exists in phylogenetically basal mammals (monotremes) and non-mammalian vertebrates [6], do not have a defined prodomain (Figure 1).

The caspase family of proteins comprises caspase-1 through -18 and the pseudo-caspase CFLAR, also known as FLIP, in mammals [6]. Notably, caspase-11 is the murine ortholog of human caspase-4 and -5 [34,35,36], and caspase-13 is the bovine ortholog of human caspase-4 and -5 [37,38]. Caspase-12 is catalytically active in some species but restricted to auto-processing or entirely inactive in others [20,33,39]. Humans lack a proteolytically active caspase-12 [40,41]. Most caspases are active in apoptosis [42,43] (Figure 1), with some of them, such as caspase-8, having additional functions as regulators of other processes [44,45]. Notably, the assignment of caspase-17 and -18 to the group of apoptotic proteases is based on sequence similarity to well-characterized paralogs, but their roles require further investigations. A subgroup of CARD-containing caspases, namely caspase-1, -4, -5 and, in some species, caspase-12, are implicated in pyroptosis, a pro-inflammatory mode of programmed cell death [46,47]. Phylogenetic analysis has shown that CARD-containing caspases (pyroptotic caspases and caspase-2 and -9) have evolved from an ancestral caspase which has also given rise to caspases without a CARD, namely caspase-14, -15 and -16 [7]. These caspases, here referred to as caspase-14-like proteases, have not been comprehensively discussed in previous reviews.

The purpose of the present article is to provide a basis for further studies into the functions of caspase-14 with potential implications on human epidermal biology and dermatology and the roles of caspase-15 and -16 in non-human species. Another aim of this review is to highlight molecular features of caspase-15 and -16, which expand the spectrum of structures and possibly the mechanisms of regulation of caspases in general.

## 2. Caspase-14: Proteolysis in Mammalian Epidermis

Caspase-14 was originally identified and investigated in three studies that could not assign a function to it [29,30,31]. The unusually short prodomain of caspase-14 and the absence of an involvement in apoptosis pointed to an unconventional role for caspase-14 [29,30,31]. Later studies revealed that caspase-14 is specifically expressed in epidermal keratinocytes, with additional minor expression in epithelial cells of the murine esophagus and thymus [48,49,50,51,52]. The transcription of the *CASP14* gene is upregulated during the terminal differentiation of epidermal keratinocytes, and the proform of the caspase-14 is activated when keratinocytes undergo cornification [48,49,53,54,55,56]. *CASP14* is also expressed during the embryonic development of the epidermis [30,57,58]. Notably, the expression of caspase-14 is associated with a mode of cell death, named cornification or corneoptosis [59,60], which is different from the caspase-dependent cell death modalities of apoptosis and pyroptosis [61,62].

Studies of *Casp14* knockout mice [63] and human patients with *CASP14* mutations [64] have shown that caspase-14 contributes to the protective function of the skin. Mechanistically, this role of caspase-14 is at least partly achieved by the proteolytic processing of filaggrin, a skin barrier protein. Filaggrin aggregates keratin intermediate filaments during cornification [65,66,67,68], and filaggrin-derived histidine is enzymatically converted into urocanic acid (UCA), a major ultraviolet B (UVB)-absorbing molecule of human skin [63,69,70]. Caspase-14 cleaves filaggrin [70,71] to control the production of free amino acids in corneocytes. Targeted deletion of *Casp14* leads to an increase in transepidermal water loss, incomplete processing of filaggrin, a decline in filaggrin breakdown products such as UCA and an increased sensitivity to UVB irradiation in mice [63,72,73]. Human patients with a rare frameshift mutation of *CASP14* develop autosomal recessive congenital ichthyosis 12 (ARCI12) [64]. These patients entirely lack caspase-14 and exhibit non-erythematous fine whitish scales on their skin, all over the body [64].

Notably, caspase-14 is conserved in monotremes (echidna and platypus), whereas filaggrin-related proteins of monotremes display little sequence similarity to murine and human filaggrin. This lack of co-evolution raises the question as to whether caspase-14 targets other substrates in these phylogenetically basal mammals [74]. Human caspase-14 was reported to cleave and activate mesotrypsin, also known as serine protease 3 (PRSS3), which in turn activates saposin, a regulator of the intercellular lipid-dependent permeability barrier of the epidermis [75]. Furthermore, caspase-14 was reported to cleave the inhibitor of caspase-activated DNase (ICAD) to allow the entry of caspase-activated DNase (CAD) into the nucleus of terminally differentiated keratinocytes [76]. Caspase-14 deficiency predisposes mice to parakeratosis, an impairment of nuclear breakdown, in the imiquimod-induced psoriasis-like model [77]. Further studies are required to stringently test the proposed substrates of caspase-14 and to screen for potentially unknown substrates.

The control of activation and catalytic activity of caspase-14 is unusual among caspases. The proteolytic separation of the large and small subunit, which is essential for activation, is not mediated auto-catalytically or by other caspases at an aspartic acid residue. Rather, an as-yet-unknown protease cleaves human procaspase-14 between isoleucine 152 and lysine 153 [78,79]. These two residues are not conserved in caspase-14 homologs of other species [80,81]. However, the presence of hydrophobic amino acid residues on the amino-terminal side of the human cleavage site is conserved, suggesting that caspase-14 could be processed by an elastase-like serine protease [80]. Kallikrein 7, a protease abundant in terminally differentiated keratinocytes, was implicated in the proteolytic processing of caspase-14, leading to the carboxy-terminal truncation of the large subunit [82]. At the amino-terminus, caspase-14 is cleaved after an arginine (R) residue [80], suggesting proteolysis by a trypsin-like enzyme.

**Figure 2:**
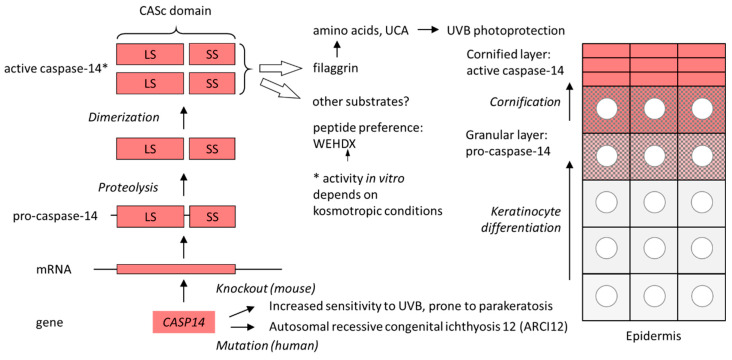
Expression, activation and function of caspase-14. The schematic on the left shows the path from the *CASP14* gene to the proteolytically active caspase-14. The schematic on the right depicts the cellular organization of the human epidermis. Keratinocytes move toward the skin surface (top) as they undergo differentiation. Pro-caspase-14 is converted into active caspase-14 in parallel with the histologically detectable degradation of the nucleus (circle) in the course of cornification. *, the activity depends on kosmotropic conditions in vitro. LS, large subunit; SS, small subunit; UCA, urocanic acid; UVB, ultraviolet B.

Proteolytic cleavage in the catalytic domain is followed by the dimerization of processed caspase-14, which is required for proteolytic activity [53,83]. Caspase-14 requires kosmotropic salts for proteolytic activity in vitro [83]. It is possible that the “order-making” conditions of kosmotropic solutions of salts such as sodium citrate mimic the milieu of the cornified layer of the epidermis, the site of caspase-14 activity in vivo [53] (Figure 2). The peptide sequence preference of isolated caspase-14 (WEHD) corresponds to that of pro-inflammatory caspases [83,84].

Caspase-14 is constitutively expressed and activated in the uppermost layers of a normal epidermis [48,49,50], whereas its expression is altered in diseased skin [81]. In psoriatic skin and in lesions of atopic dermatitis, caspase-14 is diminished [85,86,87]. In a mouse model of atopic dermatitis, allergic sensitization is not controlled by caspase-14 [88]. The expression of caspase-14 is elevated by a vitamin D3 analog clinically used for the topical treatment of psoriatic lesions [85]. Likewise, ceramides enhance caspase-14 expression [89]. By contrast, retinoic acid impairs keratinocyte differentiation and decreases the expression of caspase-14 [85,90]. A lack of caspase-14 leads to a mild imbalance in microbiome composition on the skin surface of mice [91]. The expression of caspase-14 is dysregulated in various types of cancer, leading to the suggestion that it might be a clinically useful a biomarker [92,93,94,95]. However, this concept remains to be further tested and validated. A caspase-14-derived peptide, RGEQRDPGETVGGDE, was recently identified as the target of antibodies which can be used as diagnostic biomarkers for rheumatoid arthritis in patients negative for anti-cyclic citrullinated peptide antibody and rheumatoid factor [96].

Caspase-14 is the prototypical member of the subfamily of caspase-14-like proteases and the only one that exists in humans. The other members of this subfamily, caspase-15 and -16 [6] will be reviewed in the next two sections.

## 3. Caspase-15: The Only Pyrin Domain-Containing Caspase in Mammals

Caspase-15 was identified at the transcript level in a porcine embryo cDNA library [24]. Orthologs were later found in other phylogenetically diverse mammals, such as cattle, dogs, opossums and platypuses, and in reptiles such as the green anole lizard [6]. The *caspase 15* (*CASP15*) gene is located between the evolutionarily conserved genes *SLC25A38*, previously named *FLJ20551*, and *RPSA*, previously named *LAMR1*, whereas only a degenerated remnant of the CASP15 gene is present at this locus in the human genome [97]. Likewise, the main model species, the mouse, lacks caspase-15, which explains why this caspase was not identified in the early phase of research into the mammalian repertoire of caspases [98,99].

**Figure 3:**
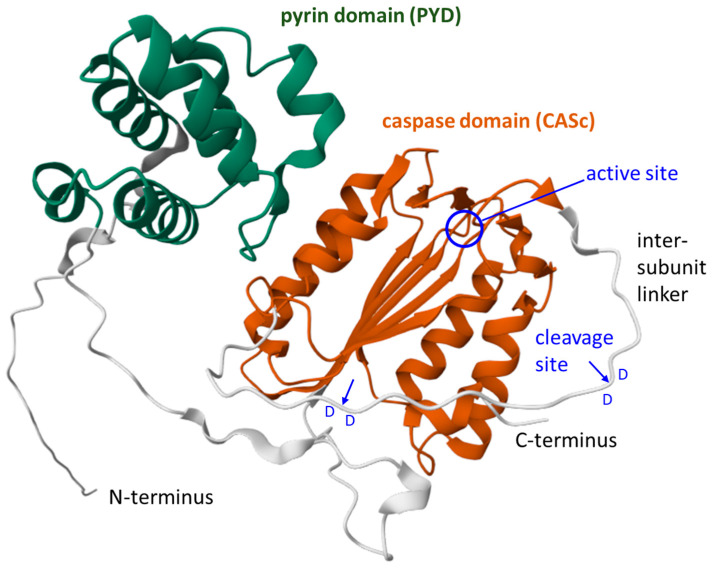
Structure model of caspase-15 of the cat (*Felis catus*). A ribbon diagram of cat caspase-15 (Uniprot accession A0A5F5XX59) downloaded from the AlphaFold protein structure database [104,105]. The pyrin domain (PYD) and the caspase domain (CASc) are highlighted. The active center (circle) of the enzyme and the cleavage sites (arrows) between aspartic acid (D) residues in the intersubunit linker are indicated. Domain 1 (PYD): CATH: 1.10.533.10; quality: high-confidence; Qscore: 85.55; boundaries: 29–111; average pLDDT: 83.20. Domain 2 (CASc): CATH: 3.40.50.1460; quality: high-confidence; Qscore: 85.26; boundaries: 147–265, 290–337; average: pLDDT 78.90. The model is a reproduction from the AlphaFold protein structure database (https://alphafold.ebi.ac.uk/entry/A0A5F5XX59, last accessed on 8 May 2025) under Creative Commons Attribution 4.0 (CC-BY 4.0) license terms (https://creativecommons.org/licenses/by/4.0/, accessed on 8 May 2025).

In contrast to caspase-14, a prodomain is present in caspase-15 (Figure 3). This prodomain is predicted to assume a pyrin fold [22,23,100]. No other mammalian caspase contains a pyrin domain, whereas at least two zebrafish caspases (caspy/caspa and caspy2/caspb), which are more closely related to caspase-1 than to caspase-15, contain a pyrin domain [25]. Further pyrin domains are present in pyrin, encoded by the *MEFV* (Mediterranean fever) gene, in the apoptosis-associated speck-like protein containing a CARD (ASC), encoded by the *PYCARD* gene, in cytosolic pattern recognition receptors of the NOD-like receptors containing a pyrin domain (NLRP), and in AIM2-like receptor families, all of which interact with members of the pro-inflammatory caspase-1 family [101]. The caspase-15 prodomain is likely to undergo homotypic interaction with one or more pyrin domains of other proteins and perhaps allows oligomerization of caspase-15 prior to activation. However, this hypothesis has not yet been tested experimentally [24]. Alternatively or additionally, the pyrin domain of caspase-15, like the pyrin domain of zebrafish caspy2, may be a binding site for lipopolysaccharide [102]. Accordingly, caspase-15 should also be tested for a potential role as a sensor for intracellular bacterial infections, similarly to human caspase-4 and -5 [36,103].

Overexpression of porcine pro-caspase-15 induces proteolytic processing into the large and small subunit and apoptosis [24]. In the course of caspase-15-induced cell death, the BH3-interacting domain death agonist (BID) is cleaved and caspase-3 is activated [24], indicating that caspase-15 is a bona fide pro-apoptotic caspase. A cysteine-to-serine mutation at the active site (C258S) prevented the catalytic and pro-apoptotic activity of caspase-15. Expression of the catalytic (CASc) domain of caspase-15 led to auto-proteolytic processing and activity toward tetrapeptide caspase substrates. The peptide sequence IETD was preferred over YVAD and WEHD [24], differing from the substrate preference of human caspase-14 (WEHD) and being more similar to that of mouse caspase-14 (IETD, WEHD) [83].

Experimental studies on recombinant porcine caspase-15 and the determination of evolutionarily conserved sequence motifs in caspase-15 of other species demonstrated that the auto-proteolytic cleavage of caspase-15 in the intersubunit linker occurs on the carboxy-terminal side of aspartic acid residues (D270 and D277) that are immediately followed by another aspartic acid residue (D271 and D278, respectively) [106] (Figure 3). The presence of an aspartic acid in the P1’ position is disfavored by other caspases [13], and autocatalytic cleavage between consecutive aspartic acid residues is very uncommon among caspases [106].

The function of caspase-15 in vivo is not known at present. The gene is expressed in several tissues but, unlike caspase-14, does not show predominant expression in the skin [24]. The presence of the pyrin domain suggests that caspase-15 is linked to the regulation of inflammation or innate immunity because pyrin domains of other proteins play equivalent roles. Given that caspase-15 is conserved in several species of domestic animals, such as pigs, cattle, horses and dogs, caspase-15 may contribute to conditions relevant to veterinary medicine.

## 4. Caspase-16: A Protease with a Caspase Domain-like Prodomain

Caspase-16 was identified as a mammalian caspase present in many but not all phylogenetic lineages. The human ortholog of the *Caspase 16* (*CASP16*) gene was originally reported as a gene called V9, which is located close to the *MEFV* gene, which encodes pyrin [107]. A cDNA derived from this gene was isolated from a cDNA library and annotated as a pseudogene transcript (“*Homo sapiens* caspace pseudogene mRNA sequence”, GenBank accession number AF098666.1; note that the erroneous spelling of this sequence name as “caspace pseudogene” is used in the database), because it did not contain an in-frame start codon close to the 5′-end. Later, orthologs of this gene were found in other mammals, leading to their annotation as *CASP16* [6]. The availability of high-quality genome sequences of many mammalian species has recently allowed researchers to determine the complete 5′-terminal portion of the *CASP16* gene in chimpanzees and other mammals [7]. Comparative analysis of the human genome revealed the first exon of the human ortholog of *CASP16* and the presence of a frameshift mutation in exon 3 [7]. These data confirmed the conclusion of previous studies [107,108] that humans have a *CASP16P* pseudogene. By contrast, *CASP16* genes of chimpanzees, rats and other mammalian species encode an apparently functional protease, caspase-16 [6,7].

Caspase-16 of mammals contains a carboxy-terminal domain of the caspase (CASc) fold in which the catalytic dyad (comprising cysteine and histidine) is conserved [7]. The linker between the large and the small subunit is longer than that in the closely related caspase-14 and -15, because the exon encoding the intersubunit linker underwent tandem duplication during the evolution of *CASP16* in placental mammals [7]. The proteolytic cleavage site in the intersubunit linker has not been determined yet.

**Figure 4:**
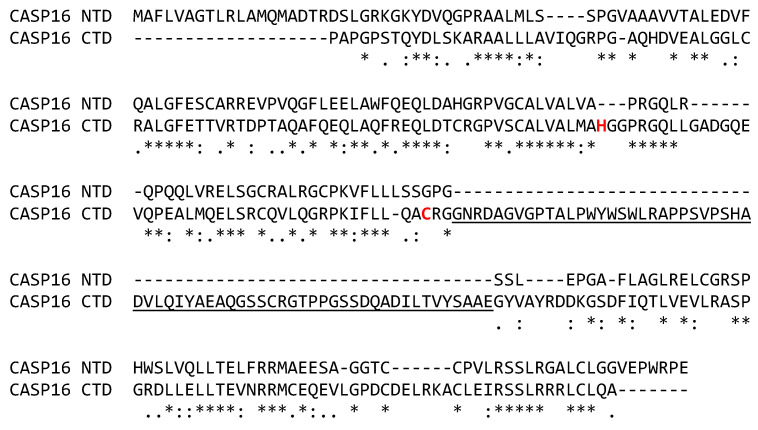
The prodomain and the catalytic domain of caspase-16 (CASP16) have similar amino acid sequences. The amino acid sequences of the amino-terminal prodomain (NTD, residues 1-204) and the carboxy-terminal catalytic domain (CTD, residues 205–470) of caspase-16 of the chimpanzee (*Pan troglodytes*), GenBank accession number XP_523278.4, were aligned. Dashes were introduced to optimize the alignment. Below the alignment, the identity, high similarity and low similarity of residues are indicated by “*”, “:” and “.”, respectively. The intersubunit linker is underlined. Amino acid residues of the catalytic dyad are highlighted by red fonts. A structure model of caspase-16 (*Macaca mulatta*) in comparison to caspase-14 (*Homo sapiens*) is available [7].

The amino-terminal domain, also referred to as a prodomain, is the most unusual feature of caspase-16, as it is predicted to assume a caspase domain-like fold (CASc). The exons encoding the prodomain are homologous to the exons encoding the large and small subunit of the carboxy-terminal (catalytic) domain, whereas no homolog of an intersubunit linker is present in the prodomain of caspase-16 [7]. Despite the significant similarity of the amino acid sequences of the prodomain and the catalytic domain, the cysteine and histidine residue critical for proteolytic activity are absent from the prodomain (Figure 4). To the best of our knowledge, no other caspase reported so far contains a CASc-like prodomain. The function and interaction partners of the caspase-16 prodomain are presently unknown. However, given that caspase domains undergo dimerization, it appears possible that an intramolecular interaction of the prodomain and catalytic domain occurs in caspase-16.

*CASP16* was reported to be expressed in the spleen and the liver of cattle [7] and in the liver of opossums [6]. According to the Pig RNA Atlas [109]), *CASP16* mRNA is highly enriched in the small intestine of the pig (*Sus scrofa*) (https://www.rnaatlas.org/ENSSSCG00000038889-na, last accessed on 14 June 2025) (Table 1). Thus, the gene expression pattern of *CASP16* may differ between species. The mechanism of activation, the substrate and the functions of caspase-16 are presently unknown. No experimental studies of caspase-16 proteins in vitro and in vivo have been reported so far.

## 5. Evolution of Genes for Caspase-14-like Proteases

**Figure 5:**
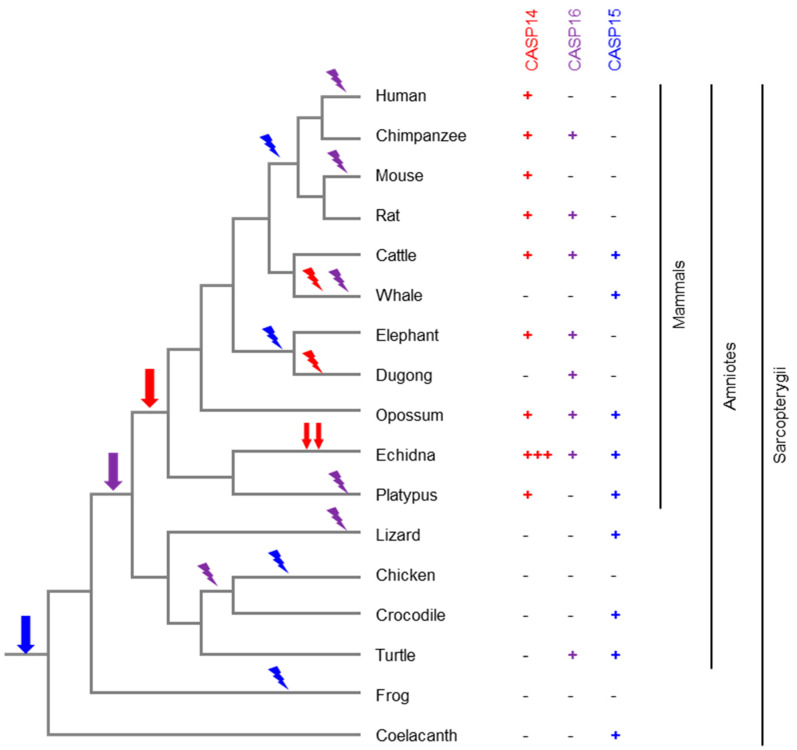
Evolution of caspase-14-like caspases in amniotes. The phylogenetic tree depicts the relationship of the species. The presence or absence of a *caspase* (*CASP*) *14, 15* or *16* gene in each species is indicated by a plus or minus symbol, respectively. Three plus symbols indicate the presence of at least three intact *CASP14* genes in the echidna. Based on the distribution of the genes in the extant species and the known phylogeny of the species [113], the evolutionary origin (arrow) and loss (flash symbol) of genes are mapped onto the tree. Red, blue and purple symbols correspond to *CASP14*, *CASP15* and *CASP16*, respectively.

The caspase-14-like proteases are linked by a common evolutionary ancestry, and the evolution of caspase-14-like genes in different phylogenetic lineages provides some hints to their potential functions. Humans have only one protein-coding caspase-14-like gene, which is *CASP14* itself (Table 1). In contrast, the closest phylogenetic relative of humans, the chimpanzee, has *CASP14* and *CASP16* [7], indicating that the pseudogenization of human *CASP16P* occurred only recently on an evolutionary timescale. According to current estimates, the two species diverged between 5.5 and 6.3 million years ago [110]. Similarly, only *CASP14* is conserved in the mouse, whereas both *CASP14* and *CASP16* are present in the rat [7] (Figure 5). Several other species of mammals have lost *CASP16*, suggesting that this gene is dispensable or even disadvantageous in some biological contexts. The list of species that have lost *CASP16* includes, but is probably not limited to, human, mouse, platypus [7], guinea pig [6] and cetaceans [111]. A correlation of *CASP16* evolution with adaptations to environmental factors or other changes in body features is not obvious. Likewise, *CASP15* is conserved in phenotypically diverse species, such as cattle, whales and opossum, whereas it has been lost in *Euarchontoglires* (including primates and rodents) and *Afrotheria* (including elephants and sirenians) [6]. The loss of *CASP15* and *CASP16* in multiple phylogenetic lineages is reminiscent of the convergent loss of genes implicated in the control of anti-pathogen responses [112]. It is conceivable that caspase-15 and caspase-16, like the classical pro-inflammatory caspase-1, -4, -5, and -11, have roles in innate immunity against microbes or viruses.

The evolution of *CASP14* is largely correlated with the adaptation of the main expression site, the epidermal barrier. The latter has changed in a profound way in whales and dolphins (together constituting the order *Cetacea*), resulting in a dramatically thickened and incompletely cornified epidermis. In line with the underlying changes in the keratinocyte differentiation program, *CASP14* has been lost in cetaceans [111]. Surprisingly, the substrate of caspase-14, filaggrin, has been retained in dolphins, indicating that at least one of the roles of filaggrin does not strictly depend on caspase-14 [110]. Similarly, *CASP14* has been lost in the fully aquatic dugong, whereas its relative, the manatee, has retained *CASP14* [113]. Intriguingly, the evolution of filaggrin paralleled that of *CASP14* in the dugong and manatee [114]. In the echidna, *CASP14* has undergone a series of duplications leading to at least three genes in which all critical sites are conserved [74] (Figure 5), but the individual functions of these paralogs are not known.

Although caspase-14 exists only in mammals, its confinement to mammals is not a shared feature of all members of caspase-14-like proteases. Members of the caspase-14-like protease family are present in non-mammalian species, indicating that the first caspase-14-like gene originated, presumably by the duplication and modification of another caspase gene, before mammals appeared in evolution. An ortholog of caspase-15 was identified in the green anole lizard [6]. Later, a caspase of the coelacanth, encoding so-called “caspase-14-like isoform X1 [*Latimeria chalumnae*]”, GenBank accession number XP_064414347.1, was found to cluster with bovine caspase-15 in a phylogenetic analysis [32] (Figure 5), indicating that it is an ortholog of caspase-15.

To evaluate whether further species need to be included in future investigations of the evolutionary origin of the caspase-14-like family of proteases, we reviewed the annotations of caspase genes in non-mammalian vertebrates available in the current version of NCBI GenBank (accessed on 7 May 2025). Erroneous annotations, such as “CASP14 caspase 14, apoptosis-related cysteine peptidase [*Gallus gallus* (chicken)], gene ID: 776274, updated on 3-Dec-2024, also known as CASP17” (https://www.ncbi.nlm.nih.gov/gene/?term=776274, accessed on 7 May 2025), corresponding to a *CASP17* gene reported previously [6], were excluded from these considerations. Caspase-14-like proteins, presumably orthologous to caspase-15 (e.g., caspase-14-like [*Dermochelys coriacea*], GenBank acc. nr. XP_038247713.1) and caspase-16 (e.g., “caspase-14 isoform X1 [*Dermochelys coriacea*]”, GenBank acc. nr. XP_038260642.1), exist in turtles, suggesting that the diversification of caspase-14-likes began before the appearance of mammals. Strikingly, caspase-14-like proteases are also predicted to be present in teleost fishes (e.g., “caspase-14-like [*Salmo salar*]”, GenBank acc. nr. XP_045567223.1), cartilaginous fishes (e.g., “caspase-14-like [*Chiloscyllium punctatum*]”, GenBank acc. nr. XP_072415151.1) and even jawless vertebrates (e.g., “caspase-14-like [*Petromyzon marinus*]”, GenBank acc. nr. XP_032831976.1), whereas no caspase-14-likes have been reported for the zebrafish [32]. Interestingly, two *caspase-14-like* genes are strongly upregulated transcriptionally in the intestine of the rainbow trout (*Oncorhynchus mykiss*) during the early defensive response to infection with the myxozoan parasite *Ceratonova shasta* [115]. The caspase-14-like proteases of fishes remain to be investigated with regard to their phylogenetic relationships, expression patterns and functions. The available data indicate that the caspase-14-like subfamily of caspases is older than the phylogenetic clade of mammals [116], and members of this caspase subfamily have been conserved in many but not all lineages of vertebrates.

## 6. Summary and Perspectives

Based on the available evidence, we conclude that caspase-14, -15 and -16 are closely related but structurally distinct members of the caspase family. For all these caspases, the primary sequence of orthologs is known in multiple species, allowing us to define conserved and presumably important features. In terms of biochemical properties and biological function, caspase-14 is the best characterized member of this group, whereas caspase-15 is only characterized with regard to its mechanism of activation and proteolytic activity in transfected cells and as an isolated protein. The least data are currently available for caspase-16, but the unique domain organization with a caspase domain-like prodomain makes caspase-16 an interesting subject of further study. The loss of *CASP16* due to pseudogenization in the human lineage has contributed to the shaping of the specific gene repertoire of humans.

Many critical research questions pertaining to caspase-14-like proteases have remained unanswered. Given the close phylogenetic relationship with pyroptosis-associated caspases such as caspase-1, do some or all caspase-14-likes interfere with pyroptosis? Does caspase-14 cleave other substrates than filaggrin too? Which protease cleaves pro-caspase-14? What are the specific physicochemical parameters that facilitate caspase-14 activity in the cornified layer of the epidermis? Do alterations in caspase-14 activity contribute to skin pathologies in patients that do not carry mutations of *CASP14*? Which stimuli lead to the activation of caspase-15 and -16? Which proteins are cleaved by caspase-15 and caspase-16 under physiological conditions? Do intramolecular interactions of the amino-terminal caspase-like domain and the carboxy-terminal caspase domain of caspase-16 suffice to support proteolytic activity without intermolecular dimerization? Are there any mRNA variants of human *CASP16P* that do not contain the frameshift, possibly due to the skipping of the mutated exon, and allow the formation of a stable protein?

Research on caspase-14-like proteases may address the questions above in different experimental and clinical settings. One priority should be the biochemical investigation of caspase-16. The expression of recombinant caspase-16 in cultured cells to test its ability to induce cell death and cleave candidate substrates can be achieved based on protocols that have been established for other caspases. A powerful approach that has been used to determine other mammalian caspases, targeted gene deletion or mutations of the active site in the mouse [117,118], is not available for studies of caspase-15 and caspase-16, because genes for these caspases are absent in wildtype mice. Deletion of caspase-16, but not caspase-15, is possible in the rat. Specific antibodies against caspase-15 and caspase-16 are required to determine the expression pattern of these proteins in tissues and cells. Similarly to antibodies against caspase-14 [48,80,119], antibodies against caspase-15 and caspase-16 may also be useful for the detection of proteolytically processed and presumably active forms of the respective caspase. Following the comparative approach that allowed the identification of *CASP15* and *CASP16* genes in the genomes of many species, proteomic screens of a broad spectrum of species and tissues may help to define the roles of these caspases. Comparative analysis of recombinant caspase-15 and -16 in parallel with other caspases will allow us to determine the functions of their unique molecular features.

## Figures and Tables

**Table 1:** Gene loci, expression patterns and protein lengths of caspase-14-like proteases in humans and pigs.

Gene	Species	Gene Locus	ENSEMBL Accession nr.	Expression (mRNA) in Tissues *	Protein Size **
*CASP14*	Human	19p13.12	ENSG00000228146	Skin	242
	Pig	chromosome 2	ENSSSCG00000013835	Skin	242
*CASP15*	Human	3p22.1 (gene remnant) ***	n.a.	n.a.	n.a.
	Pig	chromosome 13	ENSSSCG00000022773	All tissues	368
*CASP16*	Human	16p13.3 (pseudogene)	ENSG00000228146	Spleen, small intestine, liver	n.a.
	Pig	chromosome 3	ENSSSCG00000038889	Small intestine	470

* Expression data for human and pig genes were obtained from GTex (https://www.gtexportal.org/home/aboutAdultGtex, last accessed on 14 June 2025) and Pig RNA Atlas (https://www.rnaatlas.org/ENSSSCG00000038889-na, last accessed on 14 June 2025). ** The protein size is here defined by the number of amino acid residues. *** [97]. n.a., not applicable.

## Data Availability

Not applicable.
